# Study on the Influence and Contribution Rates of Field Aging Factors on Rheological Performance of Bitumen

**DOI:** 10.3390/ma18081775

**Published:** 2025-04-13

**Authors:** Shanglin Song, Yunding Zhu, Zhen Wang, Ziyang Gao, Meng Guo, Jing Huang, Xiaoqiang Jiang

**Affiliations:** 1 Gansu Provincial Highway Development Group Co., Ltd., Lanzhou 730070, China; 18193106299@163.com (S.S.); hj1001123@163.com (J.H.); 13909380007@163.com (X.J.); 2 Scientific Observation and Research Base of Transport Industry of Long Term Performance of Highway, Infrastructure in Northwest Cold and Arid Regions, Dunhuang 736200, China; 3 State Key Laboratory of Bridge Engineering Safety and Resilience, Beijing University of Technology, Beijing 100124, China; zhuyunding@emails.bjut.edu.cn; 4 The Key Laboratory of Urban Security and Disaster Engineering of Ministry of Education, Beijing University of Technology, Beijing 100124, China; 5 Beijing Municipal Road and Bridge Building Materials Group Co., Ltd., Beijing 100176, China; wangzhen_seu2006@163.com; 6 College of Materials Science and Engineering, Nanjing Tech University, Nanjing 211816, China; 15380797925@163.com

**Keywords:** field aging, bitumen, rheological performance, contribution rates

## Abstract

Laboratory simulation of aging cannot restore the complex aging behavior of bitumen well. The objective of study is to reveal the role of various field aging factors on the aging process of virgin bitumen and SBS(Styrene–Butadiene–Styrene)-modified bitumen. The influence of different field aging factors on aging behavior of virgin bitumen and SBS-modified bitumen were discussed. To achieve this goal, three different field aging styles were used to carry out field aging of virgin bitumen and SBS-modified bitumen. The aging behavior of the two types of bitumen were characterized based on rheological performance, and the influence degree of various aging factors on the rheological performance of bitumen was quantitatively analyzed through the contribution rates. The findings indicated that field aging had an obvious influence on the rheological performance of the two types of bitumen, which showed that the complex modulus, G-R parameter and stiffness modulus increased, and creep rate decreased. The contribution rates significantly indicated the degree of influence of hot oxygen, light and other factors (wind, water, dust, etc.) on the two types of bitumen. Among them, hot oxygen was the main factor leading to the aging of two types of bitumen. The virgin bitumen was not sensitive to aging caused by light, and the aging of SBS-modified bitumen was greatly affected by light. Other field aging factors (wind, water, dust, etc.) led to the aging of virgin bitumen to a certain extent, but inhibited the aging of SBS-modified bitumen.

## 1. Introduction

Flexible pavements are the most frequently utilized type of pavement for highways globally. However, throughout its production and utilization process, bitumen is easily influenced by field aging factors. The results are that the aging of bitumen causes a decline in the bitumen’s performance and the degradation of the durability of flexible pavements [1,2,3,4]. How to optimize the durability of flexible pavements in a more targeted manner is an urgent issue. The theoretical basis for solving this problem is to deeply analyze the aging process patterns during the use of flexible pavements.

The aging of flexible pavements will greatly accelerate the destruction of the pavement and enormously influence the service life of the pavement. Researchers in the field have also performed more research on the characteristics and mechanism of bitumen aging behavior. On the basis of the test results of aging samples in the laboratory, it was universally believed that aging will harden bitumen, reduce the penetration value and increase the softening point, and rheological indices such as zero shear viscosity, fatigue factor and rutting factor would increase with the increase in the aging degree of bitumen, while non-recoverable creep compliance and phase angle would decrease. Xu et al. [5] found that after bitumen aging, complex modulus increases, phase angle decreases and rheological performance weakens significantly. Ruan et al. [6] found that aging broadens the relaxation spectrum of SBS-modified bitumen, but also causes hardening of bitumen, increased viscosity, decreased ductility and other phenomena.

Domestic and foreign researchers have been attempting to simulate the field aging of bitumen by setting appropriate aging conditions in the laboratory. The currently common methods are TFOT and PAV. TFOT simulates short-term aging during bitumen production, transportation and paving. PAV simulates long-term aging during service [7,8]. Through the simulation of hot oxygen aging in the laboratory, researchers found that constant oxidation reactions occurred in the process of thermal oxygen aging, resulting in more oxygen-containing polar functional groups and other macromolecular structures in the bitumen. At the same time, the light components of bitumen undergo volatilization and transformation, leading to an imbalance in the colloidal structure of bitumen. These changes ultimately impact the macroscopic performance of bitumen [9,10,11].

However, in actual service, bitumen was not only affected by hot oxygen. The synergistic influence of temperature, light, moisture and other factors jointly leads to the aging of bitumen [12]. As early as 1961, Traxler had already found that light has an accelerating influence on the aging of bitumen [13]. Li et al. [14] found that ultraviolet radiation could significantly alter the chemical composition of bitumen. The influence of different dominant wavelengths of UV radiation on bitumen varied. Qian conducted coupling aging tests on bitumen under different water environments and UV at different wavelengths and found that some aromatic hydrocarbons, saturated hydrocarbons and light components were transformed into asphaltene recombination fractions [15].

Indoor multi-factor aging of bitumen was achieved by adding ultraviolet radiation and water. However, laboratory simulation of aging is forced aging of bitumen, which is not exactly the same as the aging behavior of bitumen in actual use [16]. The aging of bitumen in the field environment could provide practical insights into the deterioration of flexible pavement performance [17]. Wang et al. [18] explored the aging behavior of virgin bitumen and SBS-modified bitumen through field aging. The results showed that the aging of the virgin bitumen was mainly molecular polycondensation, and the short-term aging and ultraviolet aging could not completely simulate the field aging behavior of SBS-modified bitumen. Considering that bitumen is affected by complex environments during field aging, analyzing the influence of different aging factors on the field aging behavior of bitumen will effectively promote the improvement of anti-aging technology. Therefore, Song et al. [16,19] set different field aging modes and explored the influence of different aging factors on the field aging behavior of bitumen. Although existing studies have revealed the aging behavior and mechanism of different bitumen under field aging, the differences in aging mechanisms of different bitumen types are considered, such as the decomposition of modified materials in the aging process of SBS-modified bitumen. The quantification of the influence of different aging factors on different types of bitumen and the identification of aging differences still need further research.

**Figure 1:**
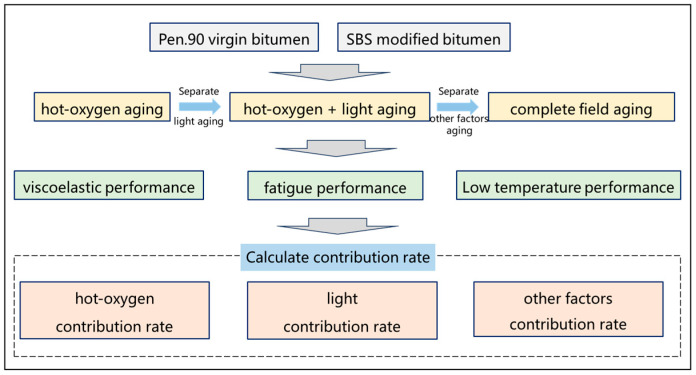
The technological roadmap.

The purpose of this study was to quantify the effects of different aging factors on the aging behavior of virgin bitumen and SBS-modified bitumen, and identify the aging differences of different aging factors on the two types of bitumen. Field aging test sites were set based on the cold and arid regions in northwest China. Samples of virgin bitumen and SBS-modified bitumen under various aging styles were obtained. Then, the viscoelastic performance, fatigue performance and low-temperature performance of virgin bitumen and SBS-modified bitumen under different field aging styles were determined by rheological experiments in order to explain the field aging behavior of bitumen. Finally, the degree of contribution of various aging factors to the rheological performance of bitumen was determined quantitatively by the contribution rates. The goal of this study was to clarify the influence of field factors such as hot oxygen, light and other field aging factors (wind, water, dust, etc.) on the aging behavior of virgin bitumen and SBS-modified bitumen and to restore the aging behavior of bitumen in a field environment. This research is helpful in deepening the aging law of bitumen in actual service and has important significance in improving the durability of flexible pavements. The technological roadmap for the paper is shown in Figure 1.

## 2. Materials and Methods

### 2.1. Materials

This study used two types of bitumen produced by Gansu Road and Bridge Shanjian Technology Co., Ltd. in Lanzhou, China, namely, Pen.90 virgin bitumen and SBS-modified bitumen obtained by modifying Pen.90 virgin bitumen. The type of SBS was linear SBS polymer. SBS-modified bitumen was prepared by high-speed shear. The specific basic physical indicators of the two types of bitumen are shown in Table 1 and Table 2. The abovementioned standard test methods come from the Ministry of Transport. The standard test methods of bitumen and bituminous mixtures for highway engineering are found in JTG E20-2011 [20].

### 2.2. Test Methods

#### 2.2.1. Field Aging Methods

Bitumen field aging tests were conducted utilizing observations and research facilities in the cold and arid regions of northwest China. The research base was located in Dunhuang City, Gansu Province. Dunhuang City has a warm temperate arid climate. The climate is dry and has less rain, the diurnal temperature difference is significant, the sunshine time is long and the ultraviolet radiation intensity is at the forefront of prefecture-level cities in China. The main meteorological data of the Dunhuang region as according to the National Meteorological Center and Dunhuang Meteorological Observation Station are shown in Table 3. The unique climatic conditions in this area create a unique aging condition for flexible pavements, which makes the flexible pavement aging process complicated and affects the longevity of flexible pavement.

**Figure 2:**
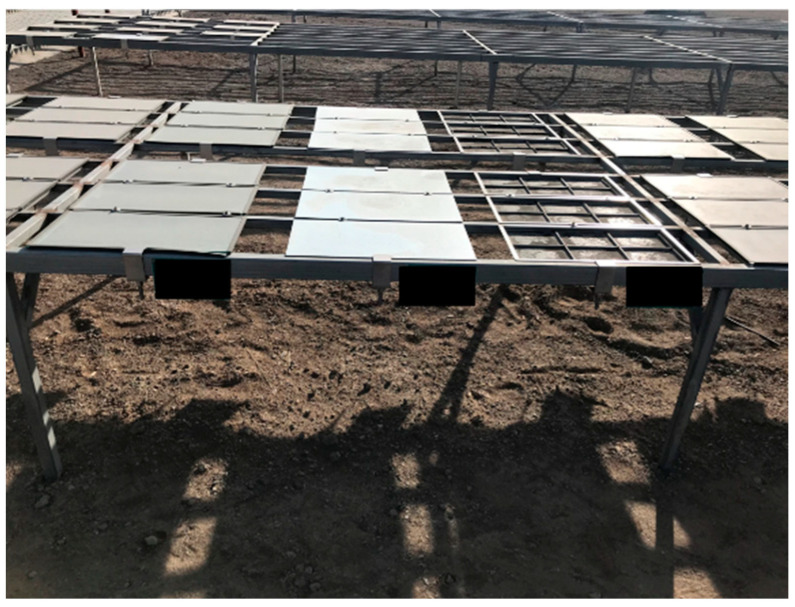
Bitumen field aging tests site.

The bitumen samples were placed on a shelf 0.5 m above the ground for field aging tests (Figure 2). The size of the sample tray was 160 mm × 115 mm. Considering the harsh aging conditions in cold and arid regions, the aging speed of bitumen will be greatly accelerated, and the aging time was set at 18 months.

**Figure 3:**
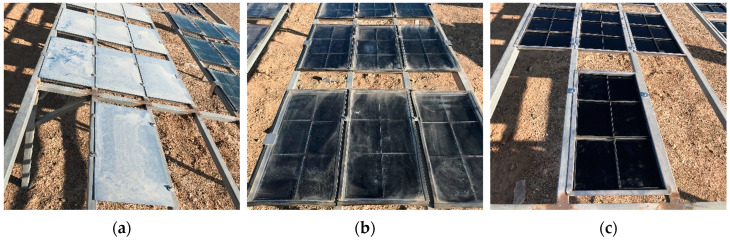
Aging styles of bitumen: (**a**) hot oxygen aging style; (**b**) hot oxygen + light aging style; (**c**) complete field aging style.

In order to explore different field aging factors’ influence on bitumen’s aging process, the bitumen samples (3.2 mm) aging were aged through three different styles following a thin film oven test (TFOT). The aging style shown in Figure 3a was a hot oxygen aging style, in which the bitumen samples were covered by an opaque plate such that bitumen aging was only affected by temperature and oxygen. This was known as O. The aging style shown in Figure 3b was hot oxygen + aging style, in which the bitumen samples were covered by a transparent plate. The impact of light on bitumen samples was increased compared to hot oxygen aging. This was known as O + L. The aging style shown in Figure 3c was a complete field aging style in which the bitumen samples were exposed directly to the environment. Bitumen samples were in direct contact with the field environment, and samples were subjected to the combined action of the field environment. It was known as All. For the hot oxygen aging style and the hot oxygen + light aging style, the distance between the bitumen surface and the covering plate was about 5 cm. There were about 128 small holes at the bottom of the device, each with a diameter of about 5 mm. In addition, there was a 5 mm gap between the periphery of the cover plate and the tray, all of which ensured that the bitumen had sufficient oxygen to undergo aging.

Due to the serious dust on the surface of the bitumen samples after field aging, 300 mL trichloroethylene was used to dissolve the bitumen samples. After the solution was completely dissolved, the dust in the trichloroethylene–asphalt solution was filtered by 15 μm, 5 μm and 1 μm filter membranes. Finally, the trichloroethylene was removed by rotary evaporation, and the bitumen samples were obtained for subsequent tests.

#### 2.2.2. Methods for Characterizing Rheological Performance

1. Viscoelastic performance characterization of bitumen.

Within this study, the rheological performance that the two categories of field aging bitumen samples represented were measured through a DSR (dynamic shear rheometer) from TA Company. The model of the test instrument is the DHR-2.

The viscoelastic performance that the two types of bitumen represented were measured with a dynamic shear rheometer. Three parallel experiments were carried out. The test used a 25 mm aluminum fixture. The range of angular frequency was established as 0.1~100 rad/s. The control mode was 1% strain mode, and the test temperatures were 35 °C, 45 °C and 55 °C.

2. Fatigue performance characterization of bitumen.

The fatigue performance of the two types of bitumen represented were measured with a dynamic shear rheometer. Three parallel experiments were carried out. The test used an 8 mm aluminum fixture. The range of angular frequency was established as 0.1~100 rad/s. The control mode was 1% strain mode, and the test temperatures were 5 °C, 15 °C and 25 °C. Then, 15 °C was used as the base temperature to construct the principal curve. The rheological parameters were obtained with a temperature of 15 °C and a loading frequency of 0.005 rad/s. Equation (1) could be used to calculate the Glover–Rowe (G-R) parameters.

(1) $$G-R=\frac{\left|G^{*}\right|{\left(\cos\delta \right)}^{2}}{\sin\delta }$$

In the formula, G-R is the Glover–Rowe (G-R) parameter (kPa);$\;G^{*}$ is the complex modulus of asphalt binder at 15 °C, 0.005 rad/s (kPa); δ is the phase angle of asphalt binder at 15 °C, 0.005 rad/s (°).

3. Low-temperature performance characterization of bitumen.

The low-temperature performance of the two types of bitumen represented were measured using a dynamic shear rheometer. Three parallel experiments were carried out. The test used an 4 mm aluminum fixture. The range of angular frequency was established as 0.2~100 rad/s. The control mode was 0.1% strain mode, and the test temperatures were −6 °C, −12 °C and −18 °C. Then, the stiffness modulus S and creep rate m values of the two types of bitumen at −6 °C, −12 °C and −18 °C were determined in order to depict the bitumen’s low-temperature characteristics.

### 2.3. Calculation Formula of Contribution Rates of Aging Factor

So as to quantitatively evaluate the influence of aging factors on the macro performance of bitumen with various aging styles, the contribution rate index $C_{i}$ of aging factors was set to quantitatively analyze the contribution of aging factors. When $C_{i}$ was less than or equal to 0, it was considered that the aging factor can reduce the i index of bitumen. When $C_{i}$ was greater than 0, it was considered that the aging factor can improve the i index of bitumen.

The contribution rate of complete field aging should be 100%. The impact of complete field aging on bitumen should not include TFOT aging. Therefore, the difference between the results of the complete field aging test and the TFOT aging test is used as the denominator, while the changes in test results caused by hot oxygen, light and other factors are used as the numerator, as shown in Equations (2)–(4). The contribution rates of hot oxygen, light and other field aging factors (wind, water, dust, etc.) to bitumen aging were calculated by Equations (2)–(4), respectively.

(2) $$C_{i-O}=\frac{G_{i-O}-G_{i-T}}{G_{i-All}-G_{i-T}}$$

(3) $$C_{i-L}=\frac{G_{i-O+L}-G_{i-O}}{G_{i-All}-G_{i-T}}$$

(4) $$C_{i-Q}=\frac{G_{i-All}-G_{i-O+L}}{G_{i-All}-G_{i-T}}$$

In the formula, $C_{i-O}$ is the contribution rate of hot oxygen to the i performance of bitumen.$\;C_{i-L}$ is the contribution rate of light to the i performance of bitumen.$\;C_{i-Q}$ is the contribution rate of other field factors (wind, water, dust, etc.) to the i performance of bitumen. $G_{i-O+L}$ is the test result of i performance after hot oxygen aging. $G_{i-O+L}$ is the test result of i performance after hot oxygen + light aging. $G_{i-All}$ is the test result of i performance after complete field aging.$\;G_{i-T}$ is the test result of i performance after TFOT.

## 3. Results and Discussion

### 3.1. Influence of Various Aging Styles on Rheological Performance of Bitumen

#### 3.1.1. Influence of Various Aging Styles on Viscoelastic Performance of Bitumen

**Figure 4:**
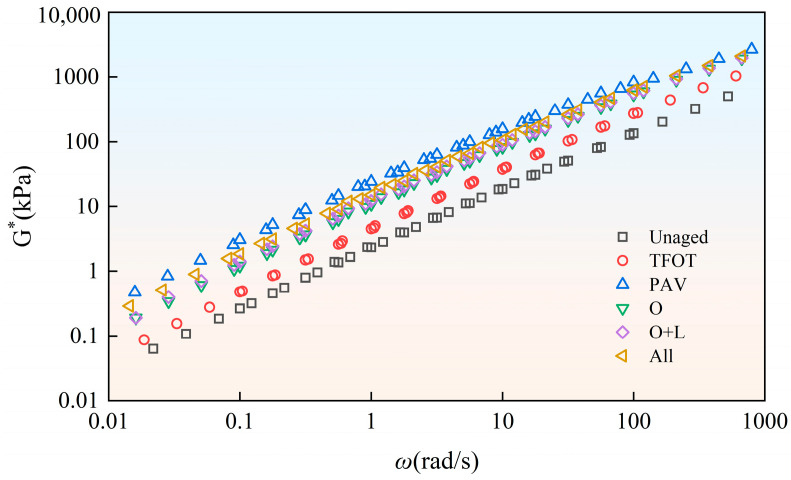
Complex modulus master curve of Pen.90 virgin bitumen after field aging.

Bitumen has obvious viscoelastic mechanical behavior. Under conditions of increased temperature and decreased frequency, bitumen tends to be viscous. Under conditions of decreased temperature and increased frequency, bitumen tends to be elastic. The temperature and frequency have a notable influence on the mechanical behavior of bitumen. On this foundation, researchers proposed the time–temperature equivalence principle. It could convert bitumen viscoelastic equivalence at different frequencies and temperatures. Utilizing the time–temperature equivalence principle, the complex modulus (G*) curves at 35 °C and 55 °C were shifted horizontally with 45 °C as the base temperature, and the principal curves of bitumen were obtained. We could thereby characterize the rheological characteristics in the wider frequency domain (Figure 4).

As depicted in Figure 4, aging in different styles increased the complex modulus of virgin bitumen. This indicated that the elastic component in the virgin bitumen was enhanced and the viscous component was weakened. The brittleness and hardness of bitumen were increased, and the capacity for anti-deformation was enhanced, but the viscoelastic property was weakened. Under various aging styles, the complex modulus of virgin bitumen showed a different increase range. The influence of indoor TFOT on the increase in the complex modulus of virgin bitumen was limited. The complex modulus of virgin bitumen showed a large increase after indoor PAV and field aging. This was because, during the aging process of bitumen, some light components evaporated, and some were converted into heavy components through chemical reactions, which made the bitumen harder [21,22,23]. In the field aging process, the complex modulus of virgin bitumen obviously increased after hot oxygen aging. This indicated that hot oxygen can promote the aging of the bitumen matrix. The addition of light and other factors (wind, water, dust, etc.) further increased the complex modulus of the virgin bitumen. However, the influence was small.

**Figure 5:**
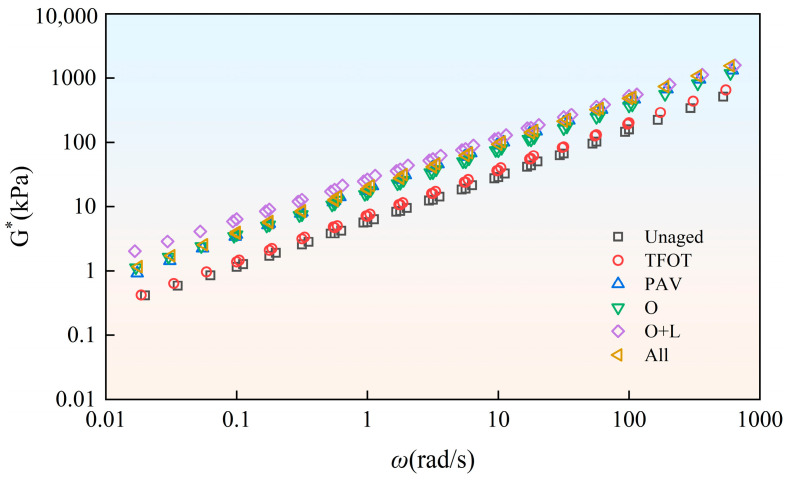
Complex modulus master curve of SBS-modified bitumen after field aging.

As depicted in Figure 5, the influence of TFOT aging on SBS-modified bitumen was very limited. However, field aging and indoor PAV had an obvious influence on SBS-modified bitumen. After hot oxygen aging, the main curve of the complex modulus of SBS-modified bitumen obviously increased. This indicated that hot oxygen had an obvious influence on the aging of SBS-modified bitumen. The addition of light also increased the complex modulus of SBS-modified bitumen. However, the influence of light aging on the complex modulus of SBS-modified bitumen was more obvious than in virgin bitumen. This was because, for SBS-modified bitumen, ultraviolet radiation in light not only affected the bitumen, but also severely destroyed the network structure in SBS-modified bitumen. This led to the degradation of SBS [24,25,26,27]. Compared with the hot oxygen + light aging style, the complex modulus of SBS-modified bitumen in the complete field aging style decreased to a certain extent. The additional field aging factors in the complete field aging style were generally considered to be wind, water, dust, etc. This meant that other factors (wind, water, dust, etc.) reduced the aging degree of SBS-modified bitumen.

**Figure 6:**
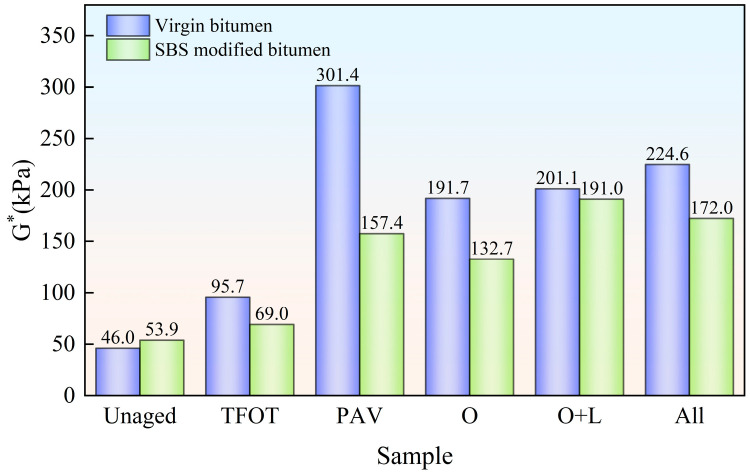
Average complex modulus of bitumen after field aging.

To gain a more intuitive understanding of the impact of different aging styles on the viscoelastic performance of virgin bitumen and SBS-modified bitumen, as well as to conduct an in-depth comparison of the effects of aging on bitumen with and without SBS, the average complex modulus was calculated based on the data in Figure 4 and Figure 5, as shown in Figure 6. It can be observed that hot oxygen aging significantly increases the average complex modulus of the virgin bitumen, with an increase of 100.4%. The addition of light and other factors only resulted in increases of 4.9% and 11.7%, respectively. The addition of the SBS modifier increased the average complex modulus of the bitumen. However, after undergoing different aging styles, the average complex modulus of SBS-modified bitumen was lower than that of virgin bitumen. Specifically, for indoor TFOT, indoor PAV, hot oxygen aging, hot oxygen + light aging and complete field aging styles, the average complex modulus of SBS-modified bitumen was 0.72, 0.52, 0.69, 0.95 and 0.77 times that of the virgin bitumen, respectively. This indicated that the incorporation of the SBS modifier mitigates the impact of aging on the viscoelastic performance of bitumen. However, the addition of light degraded the SBS, resulting in a reduction in its mitigating effect. Additionally, it could be seen that complete field aging increases the average complex modulus of virgin bitumen and SBS-modified bitumen by 134.8% and 149.4%, respectively. By comparing PAV aging with complete field aging, it was found that complete field aging had already caused the average complex modulus of SBS-modified bitumen to exceed that of PAV aging. However, the average complex modulus of the virgin bitumen was only 0.75 times that of PAV aging. This indicated that the PAV aging conducted under high temperature and pressure in the laboratory had, to some extent, amplified the mitigating effect of the SBS modifier on the decline in the viscoelastic performance of bitumen caused by aging.

#### 3.1.2. Influence of Various Aging Styles on Fatigue Performance of Bitumen

**Figure 7:**
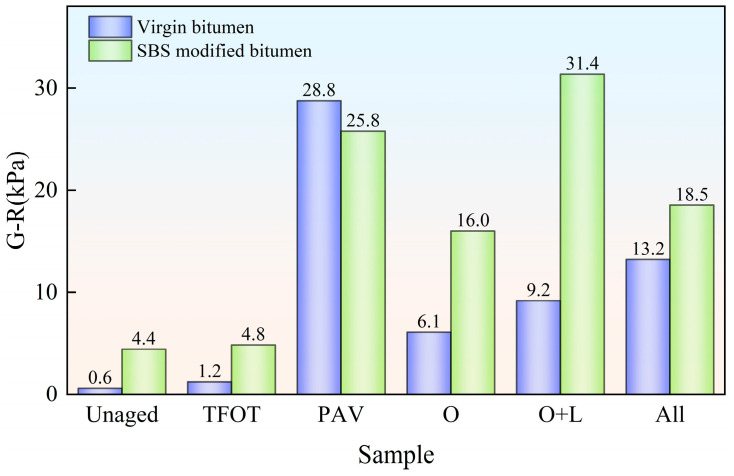
G-R parameter of bitumen after field aging.

Due to various factors affecting flexible pavements during service, there is a risk of fatigue cracking in flexible pavements [28]. So as to investigate the bitumen samples’ fatigue performance following field aging, the G-R parameter of different bitumen samples was evaluated using Equation (1), with the outcomes presented in Figure 7.

As depicted in Figure 7, the G-R parameter of virgin bitumen without aging was only 0.60 kPa. However the G-R parameter of SBS-modified bitumen without aging was relatively large, at 4.41 kPa. It could be seen that virgin bitumen had excellent anti-fatigue cracking performance, and the addition of the SBS modifier did not improve the fatigue performance of bitumen. After indoor TFOT, indoor PAV, hot oxygen aging, hot oxygen + light aging and complete field aging, the G-R parameter of virgin bitumen was increased by 102.3%, 4695.1%, 913.3%, 1428.3% and 2100.0%, respectively. It could be seen that the fatigue cracking risk of virgin bitumen is significantly magnified after aging. This was because the relaxation ability of bitumen decreased after aging. This increased the likelihood of cracking [29]. The G-R parameter of SBS-modified bitumen was increased by 9.8%, 483.8%, 262.5%, 610.5% and 319.8%, respectively. It could be seen that the addition of the SBS modifier suppressed the decline of fatigue cracking performance caused by aging. Under the three field aging styles, with the addition of hot oxygen, light, and other factors (wind, water, dust, etc.), the G-R parameter of virgin bitumen increased continuously. The risk of cracking increased accordingly. For SBS-modified bitumen, hot oxygen and light enhanced the G-R parameter of the bitumen. However, the addition of wind, water, dust and other factors reduced the G-R parameter of SBS-modified bitumen.

#### 3.1.3. Influence of Various Aging Styles on Low-Temperature Performance of Bitumen

**Figure 8:**
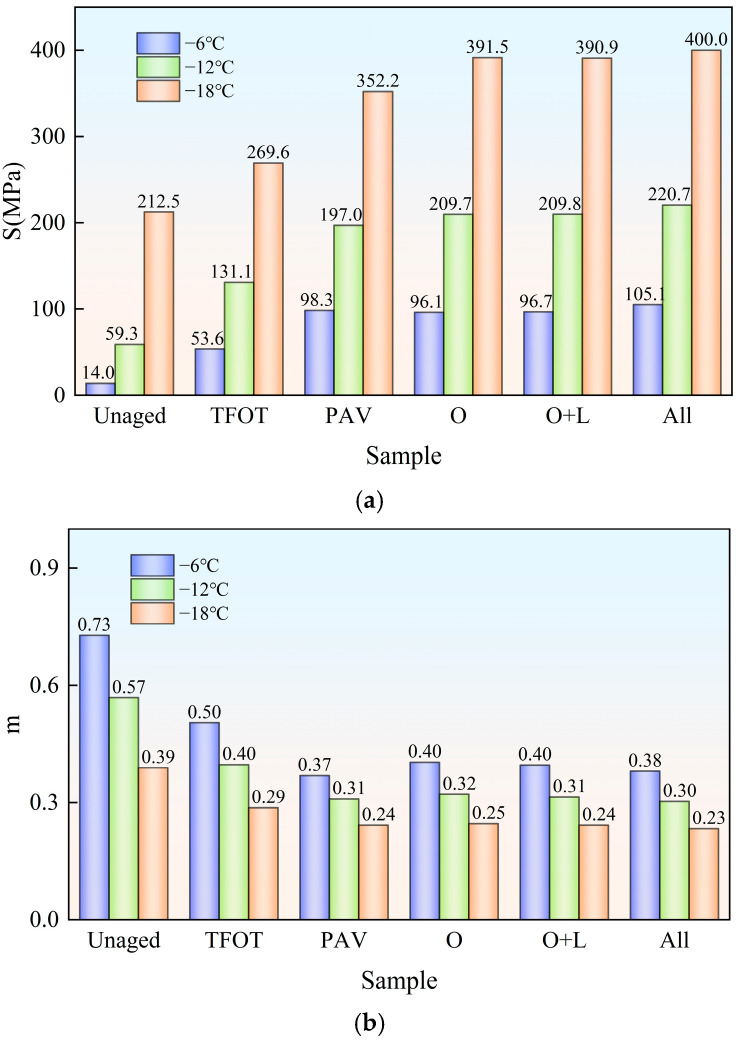
Stiffness modulus and creep rate of Pen. 90 virgin bitumen after field aging: (**a**) stiffness modulus S; (**b**) creep rate m.

**Figure 9:**
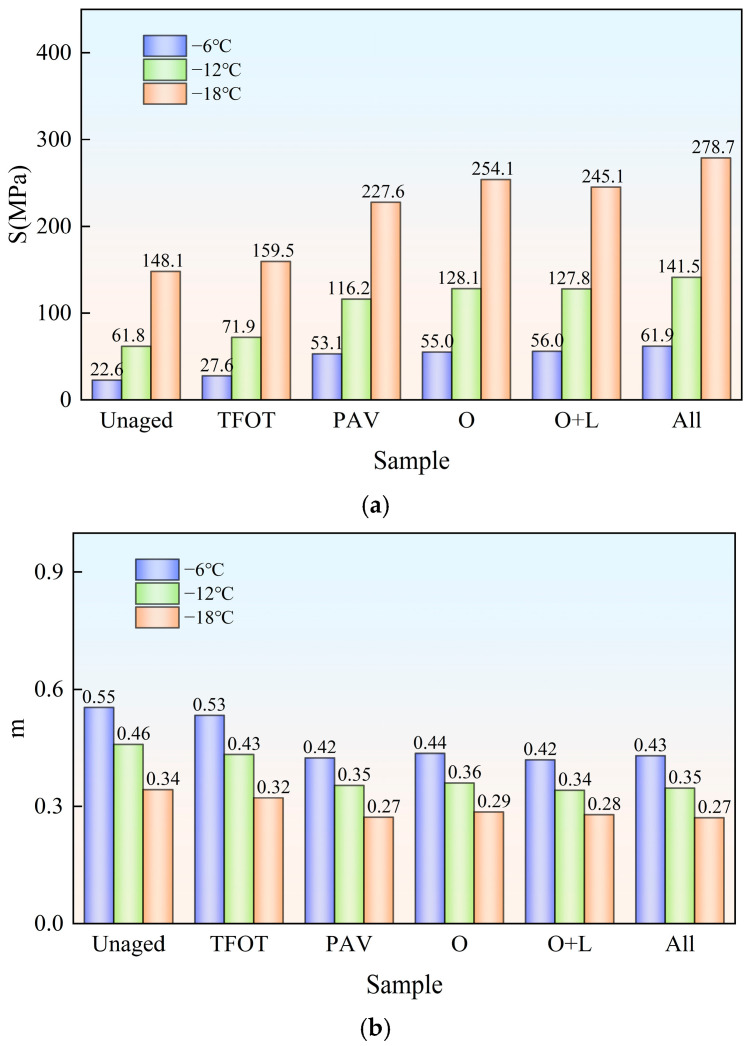
Stiffness modulus and creep rate of SBS-modified bitumen after field aging: (**a**) stiffness modulus S; (**b**) creep rate m.

Bitumen is prone to hardening under low temperatures, and it shows the characteristics of a Hooke elastomer. In Superpave, stiffness modulus S was employed as an evaluation index of bitumen flexibility. If bitumen’s stiffness modulus is large, as in bitumen at low temperatures, its creep compliance and deformation tolerance will be small. So, the low-temperature resistance will be worse. The creep rate m was utilized to indicate bitumen’s stress relaxation ability. The higher bitumen’s creep rate is, the higher its deformation capacity, the higher its relaxation ability and the better its low-temperature performance will be. The two bitumen’s stiffness modulus S and creep rate m are exhibited in Figure 8 and Figure 9.

As depicted in Figure 8, with the decrease in temperature, the stiffness modulus S of the virgin bitumen got bigger and the creep rate m decreased under various aging styles. This indicated that with the reduced temperature, the low-temperature flexibility and stress relaxation capacity of bitumen deteriorated. Subsequently, the low-temperature crack resistance of bitumen decreased. At −18 °C, the stiffness modulus S of the virgin bitumen after PAV and field aging exceeded 300 MPa, and the creep rate m value was below 0.3. None of them met the SHRP specification requirements. The stiffness modulus S of virgin bitumen was significantly improved after indoor TFOT, indoor PAV and field aging. The creep rate m decreased significantly. This showed that the aging of the virgin bitumen reduced its low-temperature cracking resistance. Among them, the effect of field aging on the low-temperature performance of virgin bitumen was more than that of PAV. During field aging, the stiffness modulus S of virgin bitumen increased by 79.2% at −6 °C due to hot oxygen aging and the creep rate m decreased by 20.0% at −6 °C. This indicated that the hot oxygen aging significantly reduced the low-temperature performance of the virgin bitumen. The addition of light and other factors (wind, water, dust, etc.) further improved the stiffness modulus S of the virgin bitumen. The creep rate m was reduced. It could be seen that light and other factors (wind, water, dust, etc.) reduced the low-temperature performance of the virgin bitumen.

As depicted in Figure 9, the addition of the SBS modifier significantly reduced the stiffness modulus S of bitumen. At −6 °C, its stiffness modulus S increased by 99.3%, 102.8% and 124.1%, respectively, due to hot oxygen aging, hot oxygen + light, and complete field aging. The creep rate decreased by 18.3%, 21.4% and 19.4%, respectively. It could be seen that hot oxygen was the main trigger for the decrease in low-temperature performance of SBS-modified bitumen. The addition of light and other factors (wind, water, dust, etc.) further reduced its low-temperature performance. By comparing the stiffness modulus of the virgin bitumen and SBS-modified bitumen at −6 °C, it was observed that after complete field aging, the stiffness modulus of both types of bitumen exceeded that of PAV aging. Specifically, the stiffness modulus of the virgin bitumen after complete field aging was 0.54 times that of PAV aging, while the stiffness modulus of SBS-modified bitumen after complete field aging was 0.59 times that of PAV aging. This indicated that PAV aging, to some extent, amplified the mitigating effect of the SBS modifier on the decline in the low-temperature performance of bitumen caused by aging.

In order to show the uncertainty range of the aging tests, the relative standard deviation was calculated based on the average complex modulus, G-R parameter and stiffness modulus of −6 °C. As depicted in Table 4, the relative standard deviations of the experiments are in a good range. Although the relative standard deviation of naturally aged samples was larger, it was still within the acceptable range.

### 3.2. Analysis of Contribution Rates of Aging Factors Under Various Aging Styles

**Figure 10:**
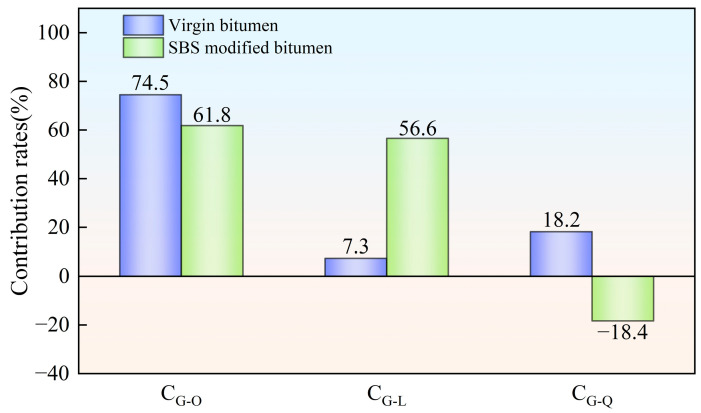
Contribution rates of bitumen aging factors based on average complex modulus.

**Figure 11:**
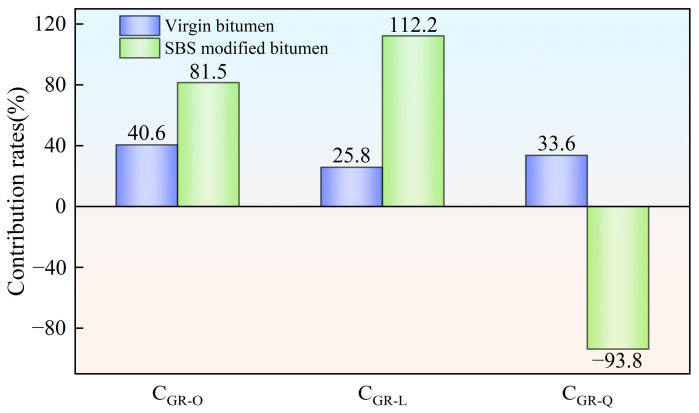
Contribution rates of bitumen aging factors based on G-R parameter.

**Figure 12:**
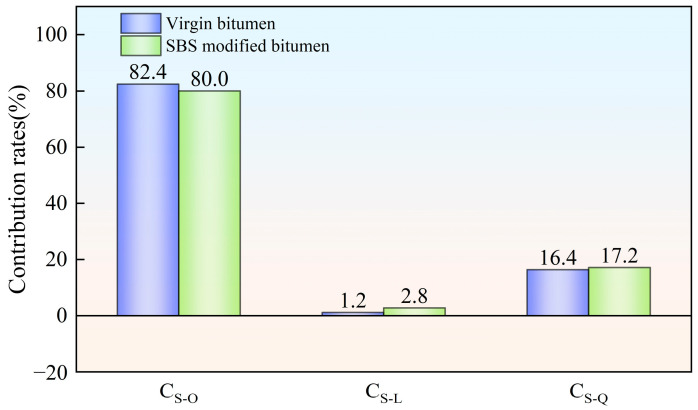
Contribution rates of bitumen aging factors based on stiffness modulus.

According to Equations (2)–(4), the contribution rates of hot oxygen, light and other factors (wind, water, dust, etc.) to the average value of bitumen complex modulus, G-R parameter of bitumen and stiffness modulus of bitumen at −6 °C were computed. The computed results are shown in Figure 10, Figure 11 and Figure 12, respectively.

Figure 10 shows directly the contribution rates of various aging factors to the average value of bitumen complex modulus. For virgin bitumen, the effect of hot oxygen on the average complex modulus was the greatest. The contribution rate was as high as 74.5%. Next was the influence of factors such as wind, water, dust, etc. The influence of light on the average complex modulus of virgin bitumen was relatively small. It could be seen that for virgin bitumen, hot oxygen had a major contribution to the increase in the average complex modulus of virgin bitumen. Unlike virgin bitumen, both hot oxygen and light had a big impact on the average complex modulus of SBS-modified bitumen. Their contribution rates were 61.8% and 56.6%, respectively. The contribution of other factors (wind, water, dust, etc.) was negative. Song et al. produced similar results [19]. This indicated that the influence of these factors on the surface of SBS-modified bitumen could decrease the negative influence of the environment on the viscoelastic performance of SBS-modified bitumen. These factors had certain anti-aging effects.

As depicted in Figure 11, in the field aging process of virgin bitumen utilizing hot oxygen, light and other factors (wind, water, dust, etc.) had certain effects on the fatigue performance of virgin bitumen. The contribution rate of hot oxygen was the highest. This indicated that hot oxygen was the main cause of fatigue performance decrease in the field aging of virgin bitumen. For SBS-modified bitumen, light played an important part in the decline of fatigue performance. The contribution of hot oxygen to fatigue degradation was second only to that of light. The influence of other factors (wind, water, dust, etc.) could inhibit the decline of fatigue performance of SBS-modified bitumen. Compared with the contribution rates based on the average complex modulus, the contribution rates of light to the fatigue performance of bitumen were significantly increased. In addition, Song et al. have found that strong light in the cold and arid northwest regions will mainly lead to an increase in microcracks on the bitumen surface [16]. This means that, on the one hand, the light reduced the fatigue cracking resistance of the bitumen while, on the other hand, it induced the cracking of the bitumen surface. It can be seen that to improve the fatigue performance of bitumen more emphasis should be placed on anti-light aging.

As depicted in Figure 12, during the field aging process, hot oxygen, light and other factors (wind, water, dust, etc.) had similar effects on the low-temperature performance of virgin bitumen and SBS-modified bitumen. Among them, hot oxygen was the main cause of the low-temperature performance decline of the two types of bitumen. Light had almost no impact on the low-temperature performance of the two types of bitumen. The contribution rates were below 3%. Wind, water, dust and other factors had a certain degree of influence on the low-temperature performance of the two types of bitumen. The contribution rates were somewhere between hot oxygen and light. The addition of the SBS modifier led to a slight decrease in the effect of hot oxygen on the low-temperature performance of bitumen. However the influence of light and other factors (wind, water, dust, etc.) on the low-temperature performance of bitumen increased.

To sum up, the calculation of contribution rates of various aging factors could more intuitively show the influence of hot oxygen, light and other factors (wind, water, dust, etc.) on the viscoelastic, fatigue and low-temperature performance of virgin bitumen and SBS-modified bitumen. The average contribution rates of different factors to the macroscopic performance of two types of bitumen were calculated from Table 5 and Table 6. It could be seen that hot oxygen was the main aging factor causing the macroscopic performance degradation of the two types of bitumen. It dominated all the parameters. This meant that the aging of bitumen under field conditions was mainly temperature-promoted oxidation reactions. The influence of light on the macroscopic performance degradation of virgin bitumen was relatively small. However, the light was more sensitive to the rheological properties of SBS-modified bitumen than the virgin bitumen. This may be related to the photodegradation of SBS-modified material. This indicated that the addition of SBS-modified material makes bitumen more sensitive to the influence of light. The stiffness modulus was almost not affected by light. This indicated that the degradation of low-temperature properties caused by aging can be alleviated by inhibiting the hot oxygen aging of bitumen. The influence of other factors (wind, water, dust, etc.) accelerates the aging of virgin bitumen. The influence of hot oxygen and light on the degradation of macro properties of virgin bitumen was between. However, for SBS-modified bitumen, the coverage role of other factors (wind, water, dust, etc.) overall restrained the degradation of its macroscopic performance. The addition of SBS-modified material increased the sensitivity to light and provided other factors (wind, water, dust, etc.) the ability to resist aging. The anti-aging research of SBS-modified bitumen is needed to synergistically optimize the anti-aging ability of hot oxygen aging, light aging, and other factors (wind, water, dust, etc.) under specific performance so as to achieve the mitigation of multi-performance degradation.

## 4. Conclusions

In this research, virgin bitumen and SBS-modified bitumen under various aging styles were obtained based on field aging. Based on viscoelastic performance, fatigue performance and low-temperature performance, the effects of various aging factors on the rheological performance of the two types of bitumen were evaluated. The macroscopic aging behavior of two types of bitumen under various aging factors was revealed. The degree of contribution of various aging factors to the two types of bitumen was quantitatively studied by calculating the contribution rates. The main conclusions were as follows:

1. The hot oxygen played a leading role in the aging process of the two types of bitumen, and the contribution rates to the rheological performance of the two types of bitumen were more than 40%. The complex modulus, G-R parameter and stiffness modulus of bitumen were significantly increased and the creep rate was reduced during the natural aging process of bitumen.
2. The two types of bitumen had different sensitivity to light. The sensitivity of virgin bitumen to light aging was low. SBS-modified bitumen showed more sensitivity to light aging, especially for its fatigue performance.
3. Other factors (wind, water, dust, etc.) show double effects on the aging of bitumen, which promote the aging of virgin bitumen. However, SBS-modified bitumen showed the effect of inhibiting aging for viscoelastic performance and fatigue performance. This may be related to the physical shielding effect of such factors.

This study preliminarily explored the impacts of various natural aging factors on the rheological performance of virgin bitumen and SBS-modified bitumen. In fact, considering that the aging of flexible pavement was mainly caused by the aging of bitumen, and the aggregate was almost not aging, this study will provide a reference for the aging prevention of flexible pavement. However, only 18 months of field aging time was set for this study, and more field aging time can be further set in the future to explore the field aging of bitumen in a more comprehensive way. In addition, the addition of vehicle load may further affect the aging of bitumen, and the influence of the coupling of load and aging on the rheological properties of bitumen can be considered in the future.

## Figures and Tables

**Table 1:** Basic physical indices of virgin bitumen.

Tests		Results	Limits	Standards
Penetration (25 °C, 100 g, 5 s)/0.1 mm		81.5	80~100	T0604-2011
Softening point (R and B)/℃		45	≥44	T0606-2011
Ductility (5 cm/min, 10 °C)/cm		100	≥30	T0605-2011
Aging tests (163 °C, 5 h)	The quality change/%	−0.13	≤±0.4	T0610-2011
	Penetration ratio/%	64	≥57	T0604-2011
	Residual ductility ratio/%	11	≥8	T0605-2011

**Table 3:** Meteorological data of the Dunhuang region.

Month	Temperature (℃)	Cumulative Value of Ultraviolet Radiation (Mj/m^2^)	Relative Humidity (%)	2 min Average Wind Speed (m/s)	Precipitation (mm)	Number of Sand Raising Days (day)
1	−6.8	10.9	42.1	1.4	0.0	0.0
2	−3.4	13.6	36.2	1.7	2.2	1.0
3	8.0	16.8	33.2	2.0	9.6	1.0
4	14.5	25.5	22.6	1.9	0.0	2.0
5	21.7	29.8	26.0	2.6	0.0	5.0
6	25.9	30.5	27.4	1.8	0.9	1.0
7	27.2	29.6	32.0	1.7	1.0	1.0
8	25.5	25.2	37.4	1.9	0.2	2.0
9	20.3	22.9	37.2	1.4	0.2	0.0
10	9.0	17.4	43.6	1.5	0.0	0.0
11	1.7	11.1	42.4	1.8	3.2	0.0
12	−8.0	9.7	48.0	1.7	0.0	0.0

**Table 2:** Basic physical indices of SBS-modified bitumen.

Tests		Results	Limits	Standards
Penetration (25 °C, 100 g, 5 s)/0.1 mm		66	60~80	T0604-2011
Softening point (R and B)/°C		87.5	≥75	T0606-2011
Ductility (5 cm/min, 10 °C)/cm		42	≥35	T0605-2011
Aging tests (163 °C, 5 h)	The quality change/%	−0.4	≤±1.0	T0610-2011
	Penetration ratio/%	75	≥65	T0604-2011
	Residual ductility ratio/%	30	≥20	T0605-2011

**Table 4:** Standard value and relative standard deviation of test data.

Samples		G * (Average)		G-R		S (−6 °C)	
		Average	RSD	Average	RSD	Average	RSD
Virgin bitumen	Unaged	46.0	4.4%	0.6	7.3%	14.0	6.3%
	TFOT	95.7	1.6%	1.2	7.0%	53.6	6.1%
	PAV	301.4	3.2%	28.8	5.2%	98.3	7.4%
	O	191.7	7.6%	6.1	9.9%	96.1	9.0%
	O + L	201.1	11.8%	9.2	14.9%	96.7	3.4%
	All	224.6	10.8%	13.2	9.1%	105.1	9.4%
SBS-modified bitumen	Unaged	53.9	4.7%	4.4	8.0%	22.6	5.3%
	TFOT	69.0	5.8%	4.8	3.8%	27.6	8.3%
	PAV	157.4	4.8%	25.8	7.6%	53.1	5.3%
	O	132.7	10.1%	16.0	11.7%	55.0	7.1%
	O + L	191.0	7.1%	31.4	13.2%	56.0	10.2%
	All	172.0	11.0%	18.5	10.2%	61.9	12.2%

“*” means the modulus is a complex modulus.

**Table 5:** Contribution rates of different factors to rheological performance of virgin bitumen.

Indices (i Performance)	Contribution Rates		
	$C_{i-O}$	$C_{i-L}$	$C_{i-Q}$
G* (average)	74.5%	7.3%	18.2%
G-R	40.6%	25.8%	33.6%
S (−6 °C)	82.4%	1.2%	16.4%
average value	65.8%	11.4%	22.7%

**Table 6:** Contribution rates of different factors to rheological performance of SBS-modified bitumen.

Indices (i Performance)	Contribution Rates		
	$C_{i-O}$	$C_{i-L}$	$C_{i-Q}$
G * (average)	61.8%	56.6%	−18.4%
G-R	81.5%	112.3%	−93.8%
S (−6 °C)	80.0%	2.8%	17.2%
average value	74.4%	57.2%	−31.7%

“*” means the modulus is a complex modulus.

## Data Availability

The original contributions presented in the study are included in the article, further inquiries can be directed to the corresponding author.
